# Developmental alterations in the transcriptome of three distinct rodent models of schizophrenia

**DOI:** 10.1371/journal.pone.0232200

**Published:** 2020-06-04

**Authors:** Jennifer J. Donegan, Angela M. Boley, Jeremy P. Glenn, Melanie A. Carless, Daniel J. Lodge

**Affiliations:** 1 Department of Pharmacology and Center for Biomedical Neuroscience, University of Texas Health Science Center, San Antonio, TX, United States of America; 2 Population Health Program, Texas Biomedical Research Institute, San Antonio, TX, United States of America; Nathan S Kline Institute, UNITED STATES

## Abstract

Schizophrenia is a debilitating disorder affecting just under 1% of the population. While the symptoms of this disorder do not appear until late adolescence, pathological alterations likely occur earlier, during development *in utero*. While there is an increasing literature examining transcriptome alterations in patients, it is not possible to examine the changes in gene expression that occur during development in humans that will develop schizophrenia. Here we utilize three distinct rodent developmental disruption models of schizophrenia to examine potential overlapping alterations in the transcriptome, with a specific focus on markers of interneuron development. Specifically, we administered either methylazoxymethanol acetate (MAM), Polyinosinic:polycytidylic acid (Poly I:C), or chronic protein malnutrition, on GD 17 and examined mRNA expression in the developing hippocampus of the offspring 18 hours later. Here, we report alterations in gene expression that may contribute to the pathophysiology of schizophrenia, including significant alterations in interneuron development and ribosome function.

## Introduction

Schizophrenia is a debilitating psychiatric disorder that affects just under 1% of the population[1]. It is characterized by positive symptoms, such as delusions and hallucinations; negative symptoms, such as blunted affect and social avoidance; and cognitive symptoms, including disruptions in working memory and cognitive inflexibility. As early as the 1980’s, it was proposed that schizophrenia was a neurodevelopmental disorder[2,3]. Evidence for this theory comes from the observation that prenatal complications such as maternal infection[4–8] and famine[9–13] significantly increase the risk of developing psychosis; and postmortem studies reveal neuroanatomical alterations indicative of abnormal neuronal development (i.e. heterotopias[14,15]). Further, many of the genes implicated in schizophrenia have been associated with neurodevelopmental processes[16,17] and the etiology of schizophrenia overlaps with the etiology of other neurodevelopmental disorders, like autism[18].

Recent work has demonstrated a key role for the hippocampus in the pathology of schizophrenia. In humans, anatomical and physiological changes are consistently observed in the hippocampus. Specifically, using imaging approaches, an increase in hippocampal activity at rest has been observed in schizophrenia patients[19–22]. This increase in hippocampal activity has been correlated with the severity of positive symptoms[23], suggesting that aberrant hippocampal activity may be a key site of pathology in schizophrenia. Indeed, we have previously used rodent models to demonstrate that aberrant dopamine system function and behavioral correlates of positive, negative and cognitive symptoms of schizophrenia are directly attributable to a pathological increase in hippocampal activity[24,25].

This increase in hippocampal activity is thought to be caused by a loss of inhibitory interneuron function. In schizophrenia patients, reductions in specific interneuron subtypes have been observed in the hippocampus[26,27], and work in our lab (and others) has demonstrated that disrupting interneuron function can induce schizophrenia-like deficits in behavior[28,29]. Further, restoring interneuron function in a rodent model of schizophrenia was able to alleviate schizophrenia-like deficits[30,31]. However, these disruptions in hippocampal and interneuron function have all been observed in schizophrenia patients after the first episode of psychosis or in the prodromal period. Due to the developmental nature of the disorder, it is important to identify the neurobiological changes that occur during gestation. Therefore, we began these studies with the *a priori* hypothesis that schizophrenia-like deficits are caused by disruptions in interneuron development and migration. This hypothesis could be tested by qPCR to measure the expression of a limited number of genes involved in interneuron development and migration. However, recent technological advances have made RNA Sequencing more feasible. Therefore, in the current experiments, we use RNA Sequencing to identify changes across the entire transcriptome to determine if development disruptions affect interneuron development as well as other neural pathways.

While there are limitations to the use of individual rodent models to parallel human schizophrenia, the examination of similarities across a number of diverse models can provide critical information about alterations that may contribute to schizophrenia. Therefore, in the current experiments, we utilize three validated developmental disruption models of schizophrenia, induced by the administration of methylazoxymethanol acetate (MAM) [32], Polyinosinic:polycytidylic acid (Poly I:C) [33], or protein malnutrition[34], to examine potential overlapping alterations in the hippocampal transcriptome during embryonic development.

## Materials and methods

All experiments were performed in accordance with the guidelines outlined in the USPH Guide for the Care and Use of Laboratory Animals and were approved by the Institutional Animal Care and Use Committee of the University of Texas Health Science Center at San Antonio.

### Rodent models

**Fig 1:**
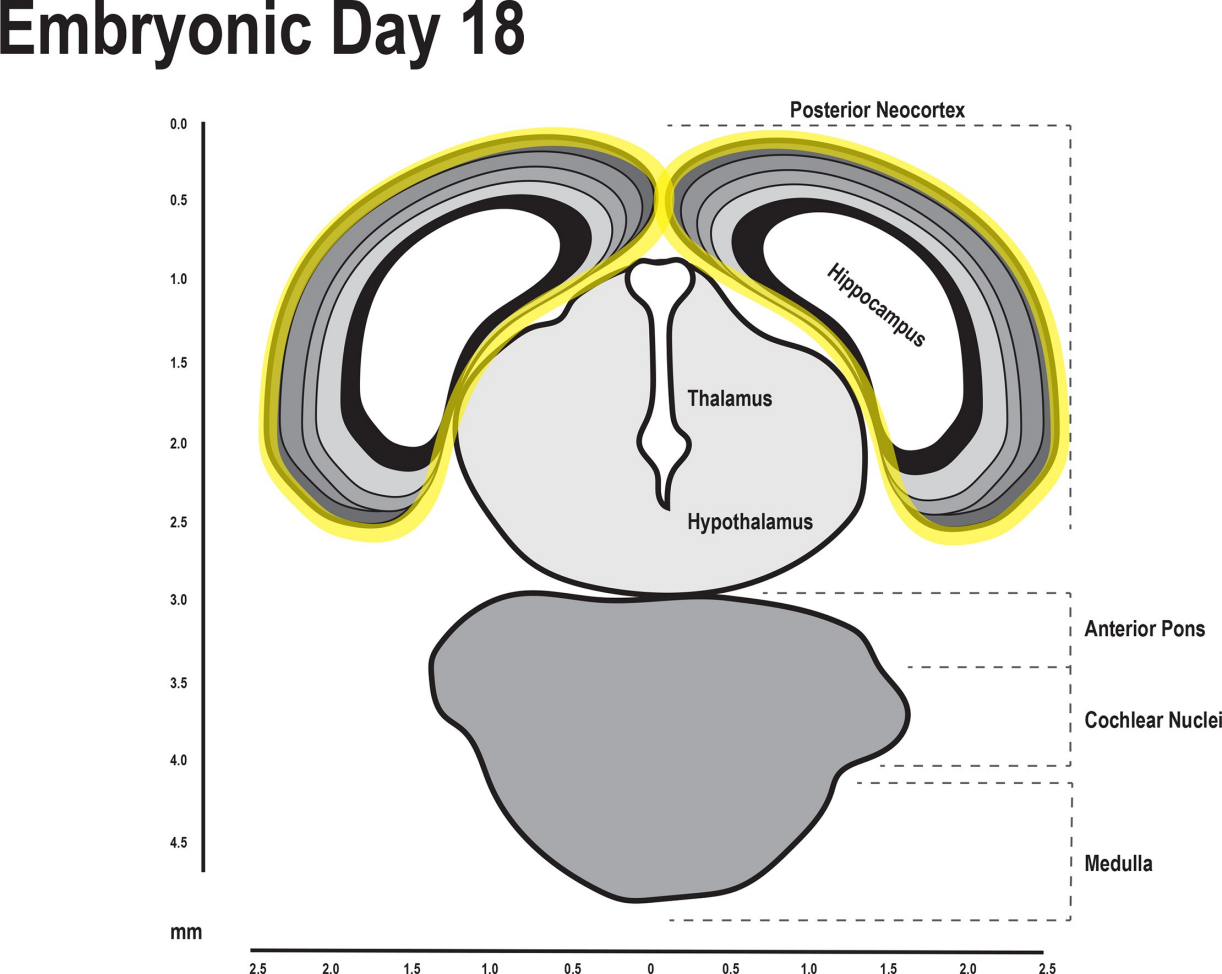
The hippocampus and neocortex was dissected for RNA sequencing. Diagram of the fetal brain on embryonic day 18. The hippocampal and neocortical regions that were dissected are outlined in yellow[35].

Timed pregnant female Sprague-Dawley rats were obtained from Envigo on gestational day 11. Either polyinosine:cytosine (Poly I:C, 7.5 mg/kg, i.p), methylazoxymethanol acetate (MAM: 22mg/kg, i.p.) or saline were administered on gestational day 17. For maternal protein malnutrition, pregnant rats had access to a low protein diet *ad libitum* (Envigo: TD.90016) containing 6.1% protein, 75.6% carbohydrate and 5.5% fat (3.8Kcal/g) from GD11-GD18. Control rats were fed an isocaloric diet of 20.3% protein, 61.6% carbohydrate and 5.5% fat (3.8Kcal/g: Envigo: TD.91352) from GD11-GD18. Pregnant rats were sacrificed on GD18 (18 hours following drug administration), three pups per mom were removed and the developing neocortex and hippocampus (Fig 1) were dissected on ice and frozen. Experiments included pups from multiple (two) litters for a total of 6 pups per group.

### RNA sequencing

RNA was extracted using the Qiagen AllPrep kit. Briefly, 17-75mg of tissue were homogenized in 600–2,400ul RLT buffer, and then processed through the AllPrep kit following manufacturer’s instructions. RNA samples were then DNase treated using the TurboDNA-free kit (Thermo Fisher Scientific) and cleaned with the Zymo RNA Clean and Concentrator kit. 250ng of RNA underwent cDNA library preparation using the Illumina TruSeq Stranded mRNA kit, followed by cluster generation and sequencing (2x100bp paired end read) on the Illumina HiSeq 2500.

In Partek Flow, input paired fastq files were trimmed by quality at both ends using phred 30 and minimum read length of 25 as cutoffs (Trim Bases tool). Quality reads were aligned to the RefSeq rn6 genome (Rattus norvegicus) with the STAR v2.4.1d aligner using default parameters for a mammalian genome. Aligned reads were quantified (Quantify to Annotation Model Partek E/M tool) against RefSeq rn6 Transcripts 80 (2017-02-06) and resulting counts were normalized by sample to TPM (Total per million) with an offset of 0.0001 (Normalize Counts tool). Normalized reads were used for GSA (Gene Specific Analysis tool) of differential expression at gene and transcript levels. For differential expression analysis, genes were included if the lowest average coverage (normalized read count) by treatment group was greater than 1.0 and if they were within the estimation reliability cutoff defined by the Partek software. In total, 5,212 genes were included for differential expression analysis.

### Quantitative polymerase chain reaction

300 ng RNA was converted to cDNA using the Applied Biosystems High Capacity Reverse Transcription Kit. Real-time quantification of diluted cDNA was performed in triplicate reactions containing sample (10 ng), Applied Biosystems TaqMan Universal PCR Master Mix (20X), and TaqMan Gene Expression Assay (20X) on a BioRad CFX384 Real Time System. Cycling Conditions consisted of one cycle at 50°C for 2 min, one cycle at 95°C for 10 min, followed by 50 cycles of denaturation (95°C for 15 sec) and elongation (60°C for 1 min). The relative gene expression was calculated using the 2^-ΔΔCT^ method. The following TaqMan Gene Expression Assays were used: Dlx1 (Rn01513884), Dlx5 (Rn00564070), Lhx6 (Rn1438474), Nkx2.1 (Rn01512482), and GAPDH (Rn01775763).

### Analysis

The RNA Sequencing data were analyzed using Advaita Bioinformatics iPathwayGuide (http://www.advaitabio.com/ipathwayguide) to define significantly affected pathways, biological processes, molecular interactions, etc. Differences between each treatment group (Poly I:C, MAM and low protein) and control were analyzed individually (S1 Table), followed by a meta-analysis of these data in order to examine potential overlapping or consistent effects of these discrete prenatal manipulations. The qPCR data was analyzed using One-way ANOVAs followed by the Holm-Sidak post-hoc test.

## Results

*All RNA Sequencing data is freely available online at the GEO (Gene Expression Omnibus) public functional genomics data repository (*https://www.ncbi.nlm.nih.gov/geo/query/acc.cgi?acc=GSE149828).

### MAM

In this experiment, **599** differentially expressed genes were identified out of a total of **5211** genes with measured expression (S1 Table). These were obtained using a threshold of **0.05** for statistical significance (p-value). These data were analyzed in the context of pathways obtained from the Kyoto Encyclopedia of Genes and Genomes (KEGG) database (Release 81.0+/01-20, Jan 17)[36,37], gene ontologies from the Gene Ontology Consortium database (2016-Sep26) [38], predicted miRNAs from the miRBase (Release 21) and MICROCOSM (Microsm version:v5) databases[39–44], and diseases from the KEGG database (Release 81.0+/01-20, Jan 17)[36,37]. In summary, **28** pathways were found to be significantly impacted (S1 Table). In addition, **503** Gene Ontology (GO) terms, **15** miRNAs (predicted based on targets), and **3** diseases were found to be significantly enriched based on uncorrected p-values (S1 Table).

### Poly I:C

In this experiment, **532** differentially expressed genes were identified out of a total of **5211** genes with measured expression (S1 Table). These were obtained using a threshold of **0.05** for statistical significance (p-value) and a log fold change of expression with absolute value of at least **0.6**. We identified **22** pathways, **527** Gene Ontology (GO) terms, **52** miRNAs, and **5** diseases that were significantly enriched based on uncorrected p-values (S1 Table).

### Protein malnutrition

In this experiment, **440** differentially expressed genes were identified out of a total of **5211** genes with measured expression (S1 Table). These were obtained using a threshold of **0.05** for statistical significance (p-value) and a log fold change of expression with absolute value of at least **0.6**. We identified **21** pathways, **476** Gene Ontology (GO) terms, **29** miRNAs, and **7** diseases that were significantly enriched based on uncorrected p-values (S1 Table).

### Meta-analysis

**Fig 2:**
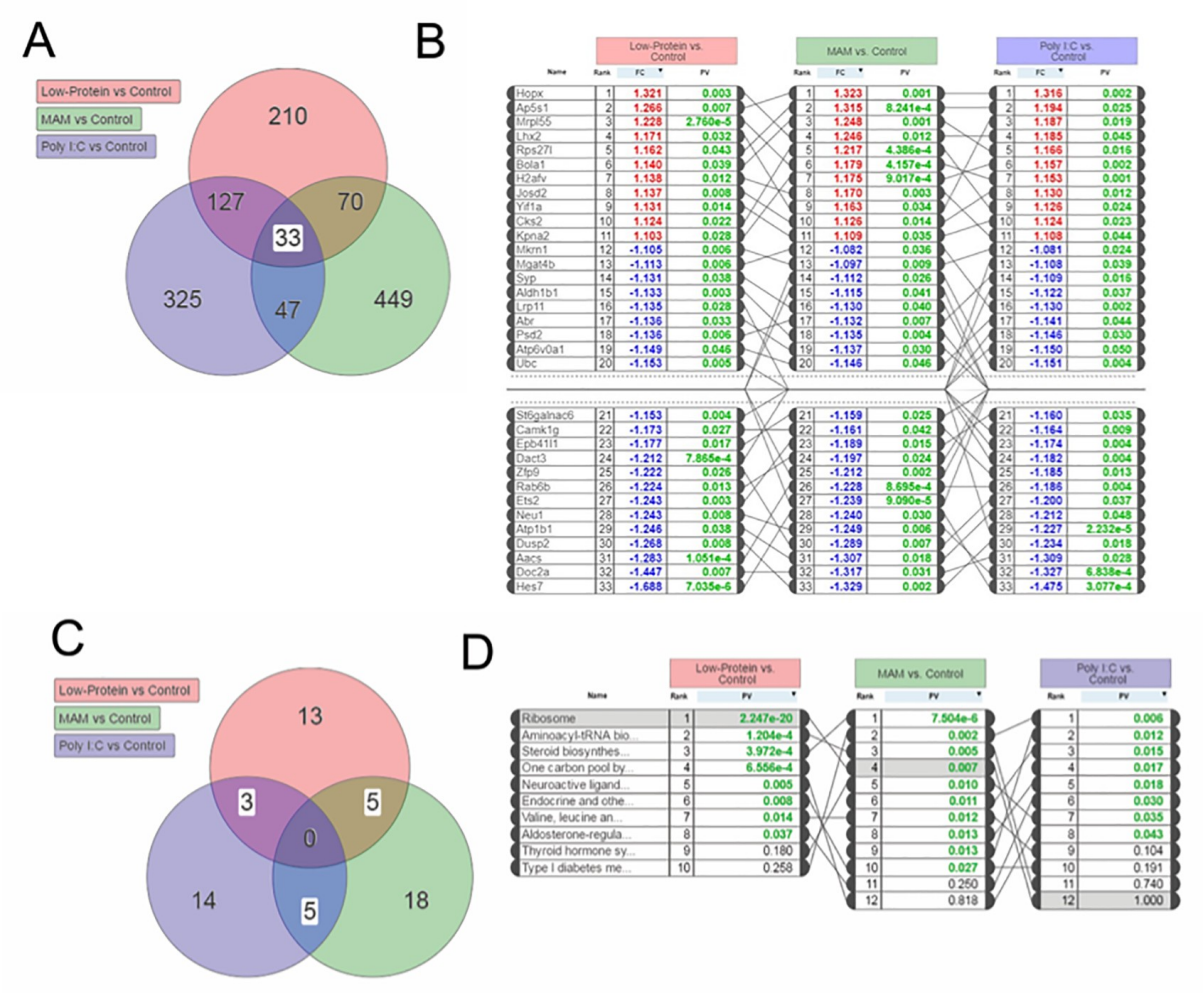
Transcriptional analysis identified consistent and overlapping changes in gene expression across three developmental models of schizophrenia. (A) Venn Diagram showing the number of genes that were differentially expressed in each schizophrenia model compared to control animals. Of these, 33 genes were differentially expressed in all three models. (B) The differentially expressed genes that were affected by all three prenatal manipulations, were ranked. (C) Venn Diagram depicting the pathways differentially altered by all three conditions. (D) Those pathways that showed overlap between at least 2 treatment groups are ranked.

**Fig 3:**
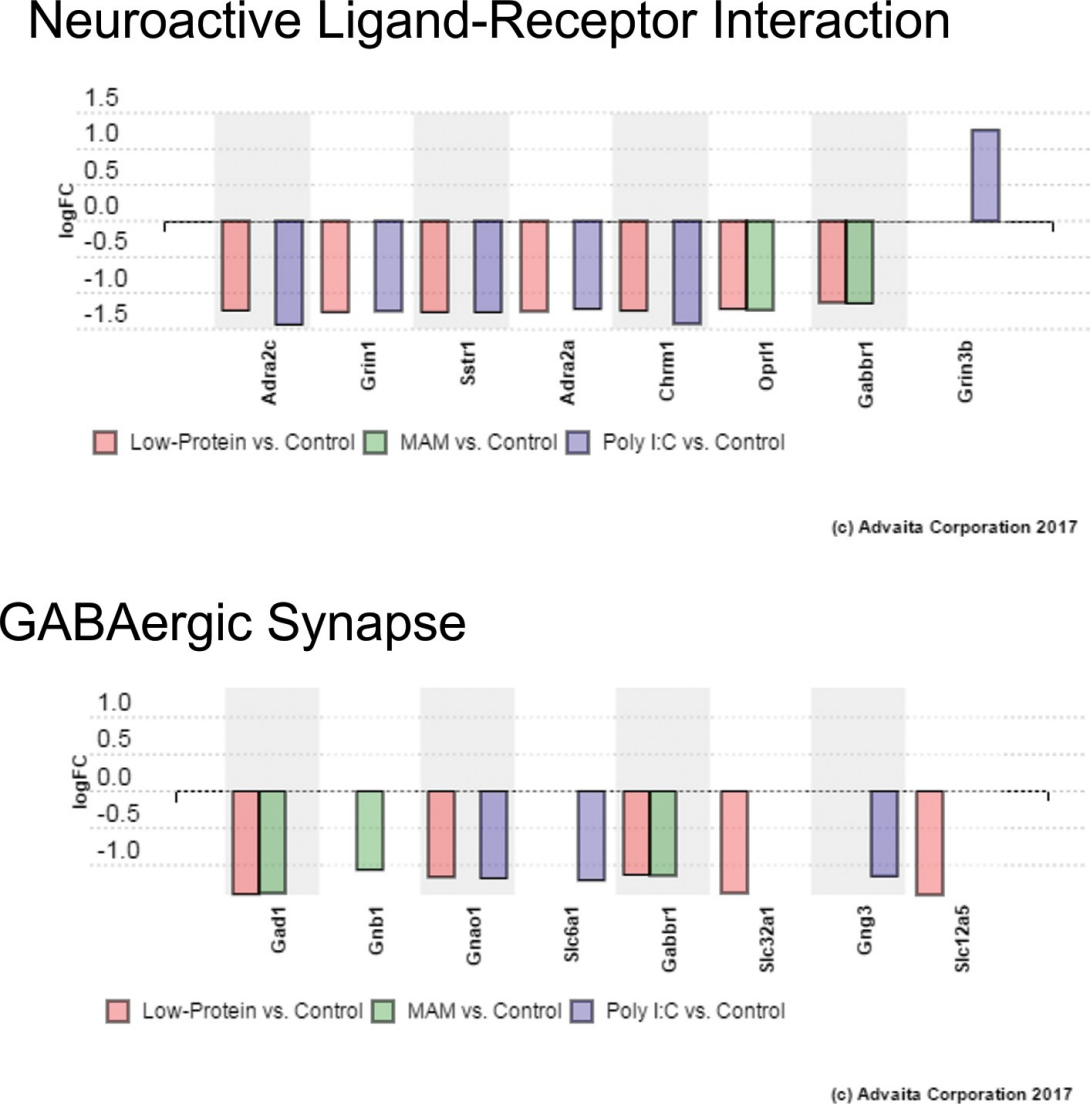
Pathway analysis identified expression changes in genes associated with Neuroactive Ligand-Receptor Interactions and GABAergic Synapses. The unbiased pathway analyses identified pathways with relevance to schizophrenia, including GO terms ‘Neuroactive Ligand-Receptor Interactions’ (ranked 5^th^) and ‘GABAergic Synapses’ (Ranked 15^th^) (A) Graph showing the differentially expressed genes in the Neuroactive Ligand-Receptor Interaction pathway. (B) Graph showing the differentially expressed genes in the GABAergic Synapse pathway.

**Fig 4:**
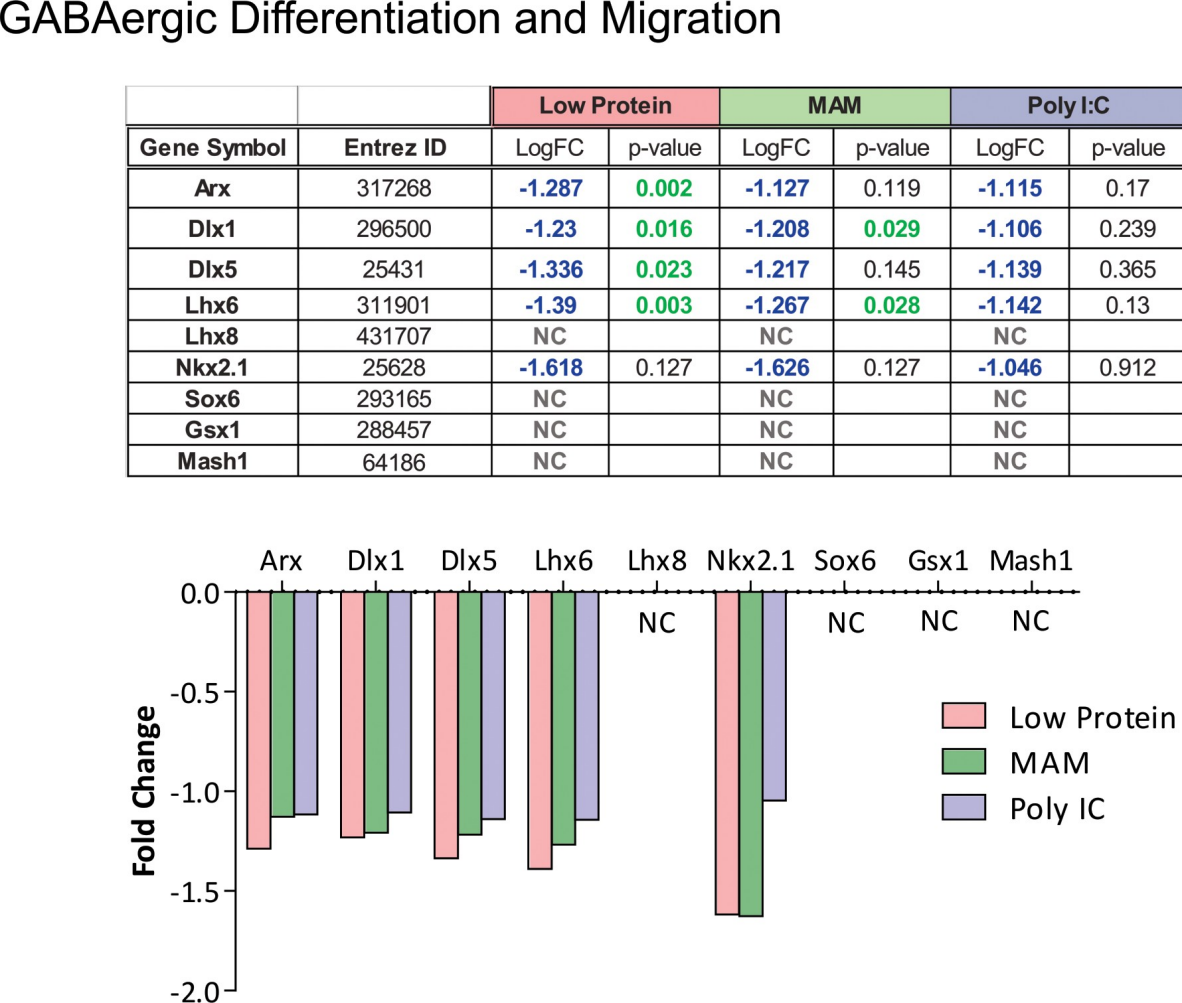
Alterations in genes associated with GABAergic development. In addition to the independent pathway analyses, we had an *a priori* hypothesis that genes involved in interneuron development and migration would be altered in these groups. Specifically, those genes associated with MGE-derived interneurons were downregulated (Arx, Dlx1, Dlx5, Lhx6, & Nkx2.1) whereas markers of CGE-derived interneurons (Sox6, Gsx1, & Mash1) were not significantly affected.

**Fig 5:**
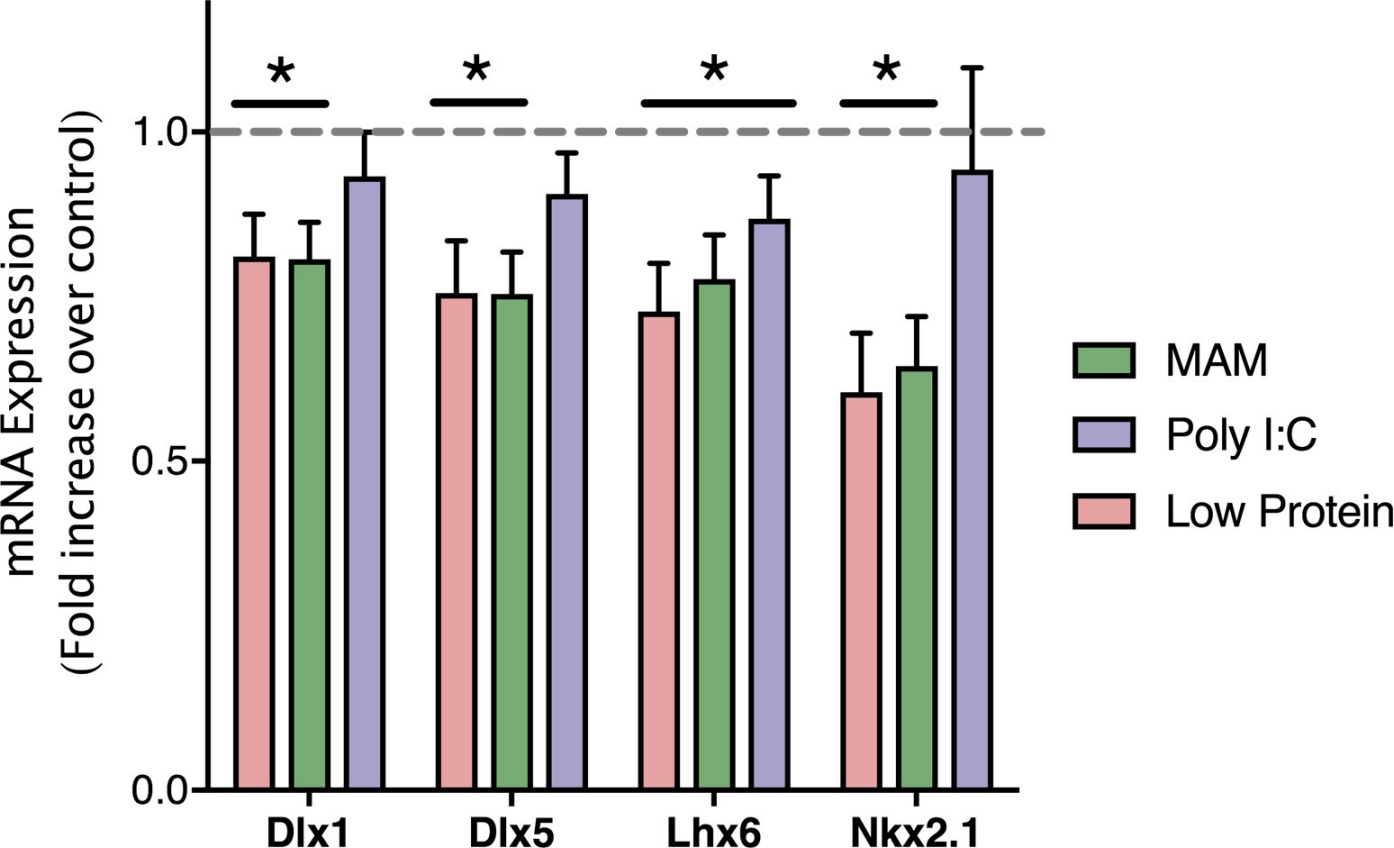
qPCR confirmation of genes associated with GABAergic development. qPCR confirmed that the expression of genes associated with the development and migration of GABAergic interneurons (Dlx1, Dlx5, Lhx6, and Nkx2.1) were decreased by developmental disruption models of schizophrenia. * is p<0.05 compared to saline-treated controls. n = 5–6 per group.

**Fig 6:**
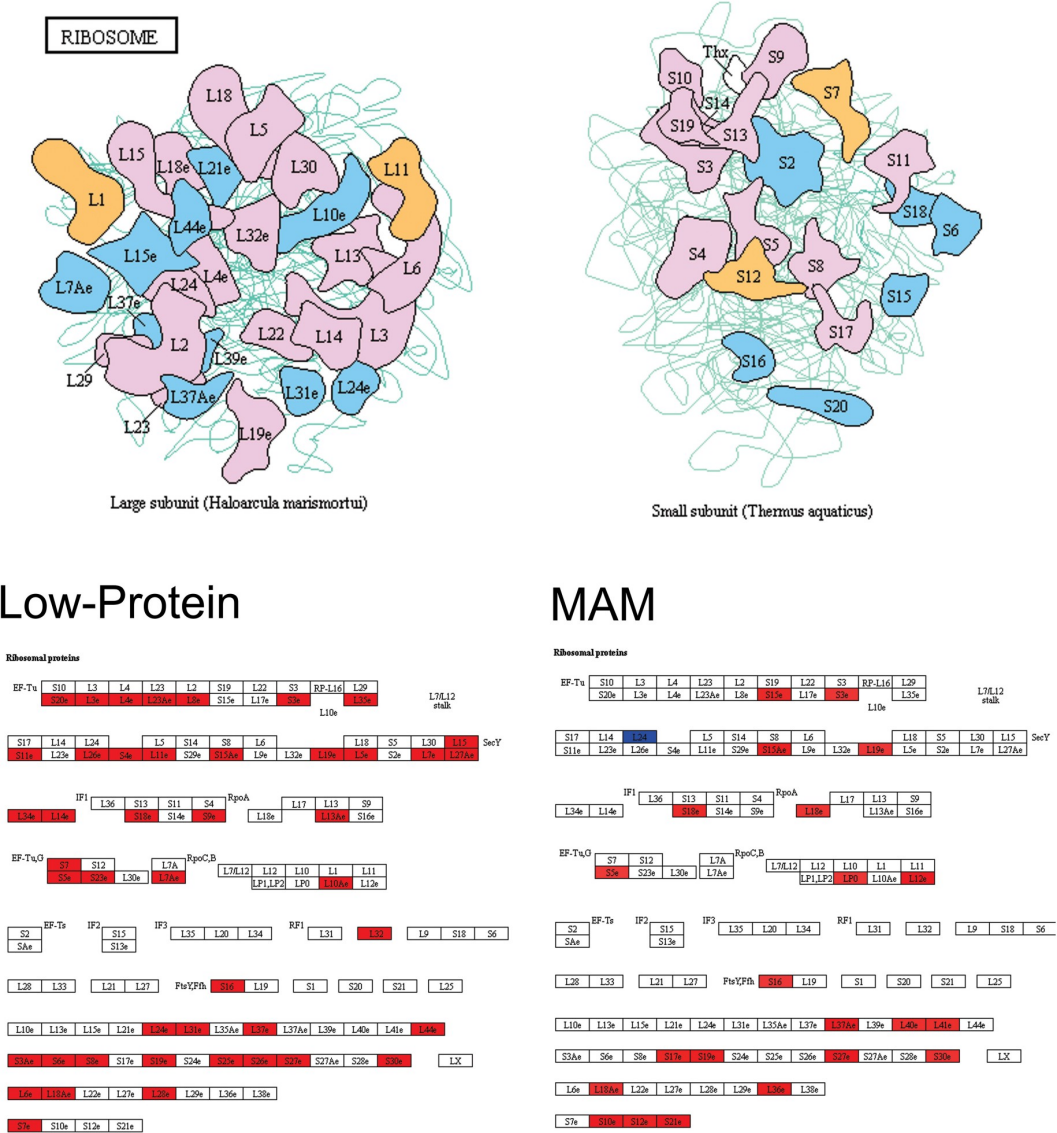
Pathway analysis identified expression changes in ribosomal proteins. The most significant pathway identified was that of Ribosomal function. (Top) Cartoon depicting proteins in the large (left) and small (right) subunits of the ribosome. (Bottom) Both Low-Protein and MAM caused changes in the expression of genes associated with ribosomal proteins. For each treatment group, an increased expression of genes encoding ribosomal proteins are highlighted in red while decreased expression is shown in blue.

To examine potential overlapping alterations in the transcriptome that could underlie the common neurophysiological and behavioral alterations observed in these rodent models, we performed a meta-analysis of these data using iPathway Guide. Of the differentially expressed genes described above, ~20% were consistently altered in 2 or more experimental conditions and 33 total genes were consistently altered across all three experimental groups (Fig 2). Similarly, 13 of the 58 pathways identified were also consistently altered in multiple (2 or more) experimental conditions (Fig 2). Of specific relevance to schizophrenia, were GO terms ‘Neuroactive Ligand-Receptor Interactions’ (ranked 5^th^) and ‘GABAergic Synapses’ (Ranked 15^th^) (Fig 3). We also had an *a priori* hypothesis that these conditions would produce alterations in genes known to regulate GABAergic neuron differentiation and migration. Indeed, we observed decreased expression of genes related to specific subsets of GABAergic development, specifically those associated with medial ganglionic eminence (MGE: but not caudal GE) derived interneurons (Fig 4). These results were confirmed using qPCR (Fig 5). Finally, some of the most robust alterations were observed in genes associated with ribosomal function (ranked 1^st^—Fig 6).

### Quantitative polymerase chain reaction

To confirm the Meta-Analysis finding that developmental disruption decreases expression of genes related to GABAergic neuron differentiation and migration, we performed qPCR. One way ANOVAs confirmed that developmental disruption significantly alters the expression of Dlx 1 (F_22_ = 10.34, p<0.05), Dlx 5 (F_22_ = 15.65, p<0.05), Lhx6 (F_22_ = 16.78, p<0.05) and Nkx2.1 (F_22_ = 14.54, p<0.05). The Holm-Sidak post-hoc analysis found that Dlx 1, Dlx 5 and Nkx2.1 expression were all significantly decreased in the Low Protein and MAM treatment groups, as compared to saline-treated controls (p<0.05). Lhx6 expression was decreased by all three developmental disruptions (p<0.05).

## Discussion

In the current experiments, we used an unbiased transcriptional analysis of the developing hippocampus and found consistent and overlapping alterations in gene expression across three distinct developmental models of schizophrenia. Because the hippocampus is hyperactive at baseline in schizophrenia patients, we chose to examine baseline transcription in the three developmental models. In the MAM model, a DNA methylating agent, methylazoxymethanol acetate (MAM), is administered to pregnant female rats on gestational day 17. This model has been shown to produce anatomical (e.g. reduced cortical thickness and hippocampal area[45]), physiological (e.g. hippocampal hyperactivity[24,30,31] and increased dopamine cell population activity[24]), and behavioral changes (e.g. stimulant-induced hyperlocomotion[24,45], prepulse inhibition deficits[45,46], latent inhibition deficits[31,47], decreased social interaction[31,46], working memory impairments[46,48], and cognitive inflexibility[31,47]) that model schizophrenia (for review, see [32]). Viral infection during pregnancy has been associated with an increased risk of schizophrenia in humans[5,49–51] and administration of the viral mimetic, polyriboinosinic-polyribocytidilic acid (Poly I:C), on gestational day 17 has also been shown to produce anatomical (e.g. enlarged ventricles and reduced hippocampal volume[52,53]), physiological (e.g. increased dopamine cell population activity[54] and increased striatal dopamine release[53]), and behavioral (e.g. stimulant-induced hyperlocomotion[53,55], prepulse inhibition deficits[55], latent inhibition deficits[53], decreased social interaction time[56,57], working memory impairments[57,58], and cognitive inflexibility[56]) deficits that resemble schizophrenia (for review, see [33,59]). Epidemiological evidence also suggests that dietary deficiencies during pregnancy is a major risk factor for schizophrenia[9,13,60–62]. Although this rodent model has been less extensively validated than MAM or Poly I:C, evidence suggests that prenatal protein malnutrition can produce anatomical (e.g. decreased prefrontal cortical volume[63]) and behavioral changes (e.g. prepulse inhibition deficits[64], decreased social interaction[65], cognitive inflexibility[66]) that model schizophrenia.

Our meta-analysis identified 33 genes that were altered consistently across the three rodent models. However, of these 33 genes, some have already been implicated in the pathology of schizophrenia. For example, the LHX2 gene encodes the Lim homeobox protein 2, a cortical selector gene that is expressed in cortical precursor cells[67]. LHX2 regulates processes such as axon guidance[68] and is required for normal hippocampal development[67]. LHX2 is down-regulated in the hippocampus of schizophrenia patients[69], while our data suggest that it is upregulated in the same region during gestation, a discrepancy that is likely explained by the developmental time point at which the gene was measured. Regardless, implication of this gene in both animal models and human patients reinforces the developmental nature of schizophrenia, and the role of the hippocampus in the pathology of this disorder[32].

In addition, we found that the Syp gene was down-regulated in all three rodent models of schizophrenia. Synaptophysin, an integral synaptic vesicle membrane protein, is encoded by the Syp gene. This result is in line with multiple human studies that have found decreased synaptophysin expression in the hippocampus and prefrontal cortex of schizophrenia patients[70–72]. Further, one study found that schizophrenia patients were more likely than controls to have rare single nucleotide polymorphisms in the Syp gene[73]. Schizophrenia patients have decreased hippocampal volumes[74], without a concomitant decrease in neuronal cell loss[75,76], leading some to hypothesize that the volume decrease is a result of reduced synaptic levels[77]. Results derived from our neurodevelopmental model of schizophrenia that show a decrease in Syp gene expression, along with similar findings in human studies, are in line with this hypothesis, as synaptophysin is a major synaptic vesicle protein and one of the most widely used markers of synaptic density.

In addition to a loss of synapses, schizophrenia has also been associated with alterations in neurotransmission. The pathway analysis performed in the current experiments identified consistent alterations (across at least 2 conditions) in genes associated with ‘Neuroactive Ligand-Receptor Interactions’ (Ranked 5^th^). Some of the genes in this pathway have already been associated with schizophrenia in humans. For example, we found that both Low-Protein and Poly I:C decreased expression of Adra2c and Adra2a, genes that encode two forms of the G-protein coupled alpha-adrenergic receptor. In humans, positive symptoms are exacerbated by noradrengergic agonists and reduced by antagonists[78]. Further, targeting the alpha 2 noradrenergic receptor has been shown to improve cognitive and negative symptoms in schizophrenia[79]. In addition, schizophrenia has also been linked to glutamatergic dysfunction. For example, antagonists of the ionotropic NMDA glutamate receptor, such as phencyclidine and ketamine, produce psychosis in humans[80]. We also found that both Poly I:C and Low-Protein treatments decreased expression of Grin1, the gene that encodes one form of the NMDA receptor. Gene association studies have identified Grin1 as a candidate gene for schizophrenia and there is a strong association between the G1001C polymorphism on the Grin1 gene promoter and schizophrenia[81]. Our results support the hypothesis that schizophrenia symptoms are associated with disruptions in neurotransmission, even during early stages of neurodevelopment.

GABA is the primary inhibitory neurotransmitter in the brain and pathway analysis identified specific changes in gene expression associated with GABAergic synapses. This is not surprising as deficits in GABAergic function have also been consistently observed in schizophrenia patients. For example, GABA is synthesized by the enzymes glutamic acid decarboxylase 1 and 2 (GAD1 and GAD2) and decreases in GAD expression have been observed across multiple brain regions, including the hippocampus and cortex[82]. This is in line with our finding that both Low-Protein and MAM treatments decreased Gad1 expression. Further, schizophrenia has been associated with altered expression of multiple GABAergic receptors. For example, a decrease in the metabotropic GABA_B_ receptor has been observed in the hippocampus of schizophrenia patients[83]. In the current experiments, we found that both Low Protein and MAM produce a decrease in expression of Gabbr1, the gene for subunit 1 of the GABA_B_ receptor. In addition, other aspects of GABAergic function are affected by schizophrenia, including transport and maintenance[84]. We also identified additional genes that were decreased by at least one of the schizophrenia models examined, including slc6a1 and slc32a1, two genes that encode GABAergic transporters, and slc12a5, the gene for the potassium-chloride cotransporter 2, which regulates intracellular chloride levels and thus GABAergic inhibition. It should be noted that in the current experiments, the dissections were not limited to the hippocampus but also included regions of the neocortex. However, the interneuron dysfunction in schizophrenia patients has been observed both in hippocampal [26,27] and cortical[85–88] regions.

We have previously demonstrated that aberrant interneuron function can induce a schizophrenia-like phenotype[28], and that transplantation of specific interneuron subtypes, derived from the MGE, can reverse neurophysiological and behavioral deficits associated with schizophrenia, in the MAM rat[30,31]. For this reason, we had an *a priori* hypothesis that developmental disruptions may alter interneuron development and migration. During development, GABAergic interneurons are born in the subpallial forebrain in progenitor regions called ganglionic eminences before migrating tangentially into hippocampal and cortical regions[89]. In these progenitor regions, the cellular fate of interneuron precursors is determined by activation of a series of morphogen-regulated transcription factors. Some of the first of these transcription factors to be activated are the Dlx homeobox genes, including Dlx1/2 and Dlx5/6. Dlx1 and Dlx2 are activated in all interneurons downstream of early patterning genes and play an important role in the generation, specification, and migration of interneurons (for review see [90]). In humans, reduced Dlx1 mRNA has been observed in the OFC[91] and thalamus[92] of schizophrenia patients. Downstream, Dlx 5/6 are direct targets of Dlx1/2, and have been shown to play a role in interneuron maturation. In the current experiments, we also found that all three prenatal manipulations decreased Dlx1 and Dlx5 gene expression, suggesting that these transcription factors may be one mechanism by which prenatal disruptions can lead to alterations in interneuron development. Specificity for the MGE developing interneurons (including PV and SST) come from the early expression of NK2 homeobox 1 (NKX2.1). Indeed, unlike the Dlx homeobox transcription factors, Nkx2.1 is absent from the LGE and CGE [93], suggesting a specific role in MGE neurogenesis. Nkx2.1 is expressed in PV and SST progenitors of the MGE and activates the downstream transcription factor, Lhx6, which is required for interneuron migration and post-migratory maturation[89]. Interestingly, all three prenatal manipulations that we examined produced a decrease in Lhx6 expression. This finding is in line with results from human studies that identified deficits in Lhx6 function in schizophrenia patients[94,95]. Further, many of the genetic cascades downstream of Lhx6 have also been implicated in schizophrenia. For example, alterations in the expression of the chemokine receptors (CXCR4 & CXCR7) have been reported [96] and an association between neuregulin signaling (via the Erb4 receptor) has been extensively studied [97–101]. Interestingly, we did not see changes in markers of pyramidal cells (CAMKII, TBR1, MAP2), astrocytes (GFAP, Slc1A3, S100B, ALDH1L1), microglia (TMEM119, CX3CR1), or oligodendrocytes (PDGFRA, OLIG2, MBP). Taken together, we posit that the decrease in Dlx1, Dlx5, and Lhx6 expression that we observed in our schizophrenia models may lead to the cell-specific deficits in interneuron development that are thought to play a key role in the pathophysiology of schizophrenia.

Finally, the highest ranked pathway observed was in ribosomal function. Specifically, we found increases in the expression of multiple genes that encode ribosomal proteins in both the Low-Protein and MAM models. Protein abnormalities have previously been observed in schizophrenia patients[102] but total protein levels can be affected by changes in a variety of factors, including transcription, mRNA stability, gene regulation by microRNAs, protein stability, and ubiquitination. However, in line with our results, others have used neural progenitor cells derived from human induced pluripotent stem cells to demonstrate that schizophrenia patients have an increase in total protein levels that was the result of an increase in translational machinery[102]. In addition, one post-mortem study demonstrated genetic changes in ribosome and translational activity in the brain of schizophrenia patients[103]. Together with our results, this suggests that the protein abnormalities associated with schizophrenia may be a direct result of increased ribosomal machinery and translation.

It should be noted, that some genes that are known to be affected in schizophrenia were not affected by all three animal models that we examined. Further, there were zero pathways that were affected by all three models. In our initial analysis, we found that 127 genes were commonly affected by protein malnutrition and Poly I:C while MAM and protein malnutrition only shared 70 common genes and MAM and Poly I:C only shared 47 common genes. Of these genes, COMTD1 has been shown to be differentially methylated in schizophrenia patients[104], which would lead to altered transcription of this gene. In the current study, we found that COMTD1 expression was significantly down-regulated by prenatal protein malnutrition and Poly I:C, but not by MAM. Conversely, the Srr gene, was only downregulated by MAM treatment. Interestingly, reduced Srr immunoreactivity has been observed in the prefrontal cortex of schizophrenia patients [105] and Srr mutant mice show schizophrenia-like behavioral deficits[106]. Although prenatal MAM, Poly I:C and protein malnutrition have all been shown to produce schizophrenia-like deficits, epidemiological evidence has only found Poly I:C and protein malnutrition to be associated with an increased risk for schizophrenia. Further, these two rodent models seem to produce less robust behavioral changes compared to the MAM model. Therefore, it is possible that MAM treatment produces schizophrenia-like deficits via a mechanism that is different than Poly I:C or protein malnutrition.

The results of the current studies are limited by the fact that only the hippocampus and neocortex were examined and that both sexes were combined. In future studies, a more nuanced approach will be used to examine transcriptomic changes across brain regions and between sexes. In conclusion, our unbiased transcriptional analysis of the developing hippocampus identified consistent and overlapping alterations in gene expression, especially related to neurotransmission, GABAergic interneuron development, and ribosomal function. Our results suggest that schizophrenia is associated with changes in gene expression that occur during gestation, and identify potential targets for future therapeutics.

## Supporting information

S1 Table (XLSX)
